# The complete mitochondrial genome sequence of the saprotrophic filamentous fungus *Umbelopsis nana*

**DOI:** 10.1080/23802359.2026.2668248

**Published:** 2026-05-07

**Authors:** Ryuka Iizuka, Tomohiro Suzuki, Yoko Katayama, Makoto Yoshida

**Affiliations:** ^a^ Institute of Global Innovation Research, Tokyo University of Agriculture and Technology, Fuchu-shi, Japan; ^b^ Center for Bioscience Research & Education, Utsunomiya University, Utsunomiya-shi, Japan; ^c^ Institute of Agriculture, Tokyo University of Agriculture and Technology, Fuchu, Japan; ^d^ Independent Administrative Institution, Tokyo National Research Institute for Cultural Properties, Taito-ku, Japan

**Keywords:** PacBio sequencing, phylogenetic analysis, mitogenome annotation

## Abstract

*Umbelopsis nana* is a saprotrophic filamentous fungus that plays a role in nutrient cycling in forest ecosystems. We determined the complete mitochondrial genome sequence of *U. nana* strain THIF13 (NBRC 117090), which is a circular molecule of 39,484 bp with a GC content of 32.7%. The mitogenome encodes 45 genes, including 20 protein-coding genes, 23 tRNA genes, and two rRNA genes. Phylogenetic analysis based on 13 Mucoromycota species and two outgroup taxa placed *U. nana* as a sister lineage to the Mucorales clade within Mucoromycotina. This study represents the first report of a complete mitochondrial genome sequence for the genus *Umbelopsis*.

## 1. Introduction

*Umbelopsis nana* (Linnem.) Arx 1984 (Von Arx 1982), a member of the order Umbelopsidales (subphylum Mucoromycotina), is commonly isolated from forest soils globally (Wang et al. 2015; Watanabe et al. 2001). *Umbelopsis* species contribute to nutrient cycling and have attracted interest for their biotechnological potential, particularly in lipid production (Chatzifragkou et al. 2010; Gardeli et al. 2017; Hou et al. 2024; Kamisaka et al. 1993; Papanikolaou and Aggelis 2019; Pillai et al. 1998). While recent whole-genome sequencing and nuclear marker analyses have expanded our understanding of this genus (Crous et al. 2017; Hou et al. 2024; Ogawa et al. 2005, 2011; Wang et al. 2014, 2015, 2022), comprehensive mitochondrial genomic resources—essential for fungal systematics and organellar evolution—are currently unavailable for the genus *Umbelopsis*. This lack of mitogenomic data limits the resolution of deeper phylogenetic relationships within the Mucoromycotina. To date, no complete mitochondrial genome has been reported for the genus *Umbelopsis*. Here, we present the first complete mitochondrial genome of *U. nana* from our recent genomic project (Iizuka et al. 2026). Through annotation and comparative analysis, this study clarifies the phylogenetic position of *U. nana* within the Mucoromycotina and provides a foundational resource for future evolutionary and functional studies of this taxonomically complex genus.

## 2. Materials and methods

### 2.1. Fungal strain and culture conditions

**Figure 1.**
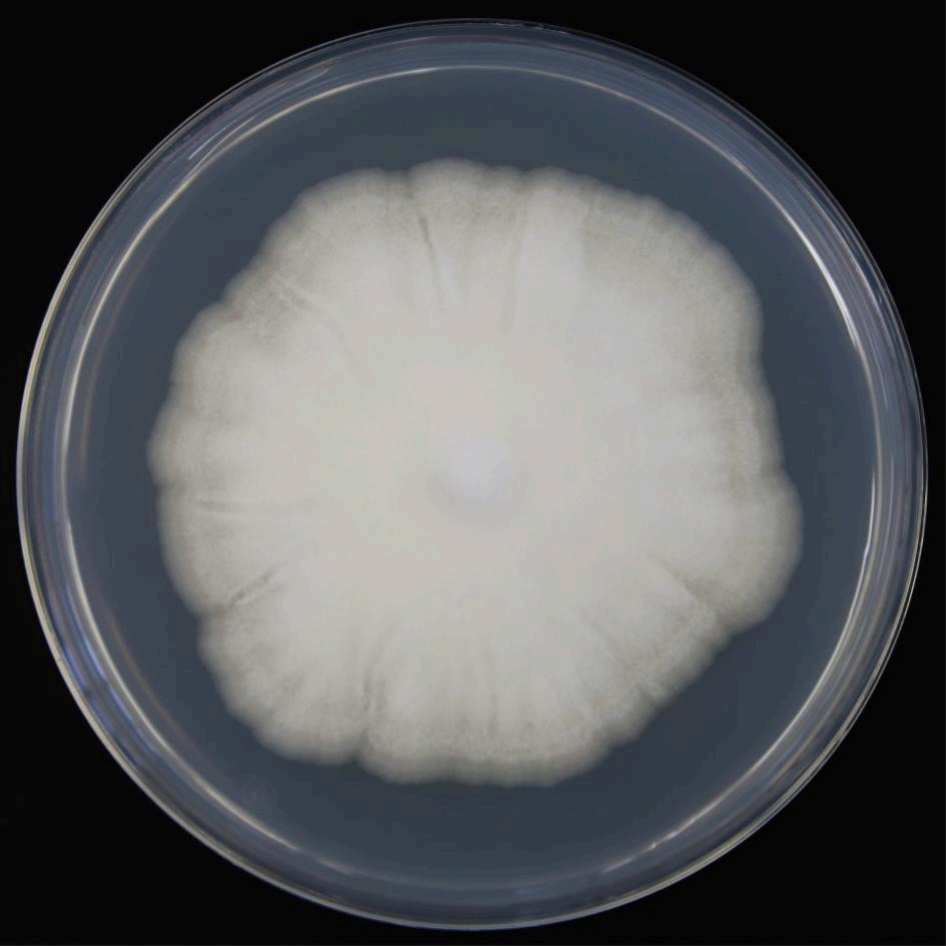
Mycelium of *Umbelopsis nana* strain THIF13 growing on PDA medium at 25 °C for 12 days. The photo was taken by Ryuka Iizuka.

*Umbelopsis nana* strain THIF13 (Figure 1), originally isolated from forest soil in the Karasawa-yama area, Tochigi, Japan (36.36° N, 139.60° E) (Masaki et al. 2016), is deposited at the NITE Biological Resource Center (NBRC; https://www.nite.go.jp/en/index.html; contact: Curator of NBRC, nbrc-order@nite.go.jp) under the voucher no. NBRC 117090. For mycelial growth, the strain was cultured in potato dextrose broth (BD, Franklin Lakes, NJ) at 25 °C with shaking at 150 rpm for 12 days. Following incubation, the mycelial biomass was collected by vacuum filtration and immediately frozen in liquid nitrogen.

### 2.2. Genome assembly and functional annotation

Total genomic DNA was isolated from the frozen mycelium using the cetyltrimethylammonium bromide (CTAB) method (Doyle and Doyle 1987). RNA was removed with RNase A (Qiagen, Hilden, Germany). A long-read library was prepared using the PacBio Microbial Library kit v3.0 (PacBio, Menlo Park, CA) and sequenced on a PacBio Sequel IIe platform. The sequencing yielded 113,886 HiFi reads (totaling 882.8 Mbp) with a minimum accuracy of 99% (Q20), characterized by an average quality of Q31 and an N50 of 9,012 bp. *De novo* assembly with hifiasm v0.25.0-r726 (Cheng et al. 2021) recovered the mitochondrial genome as a single circular contig. Initial annotation was conducted *via* GeSeq v2.03 (Tillich et al. 2017) using BLAT searches (Kent 2002) against five Mucorales reference mitogenomes (*Absidia glauca* [NC_036158]*, Lichtheimia hongkongensis* [NC_024200], *Parasitella parasitica* [NC_024944]*, Rhizopus arrhizus* [NC_006836], and *Phycomyces blakesleeanus* [NC_027730]), with tRNA genes predicted by ARAGORN v1.2.38 (Laslett and Canbäck 2008; Laslett et al. 2002). Following automated annotation, all identified genes were manually curated. For protein-coding genes (PCGs) with incomplete open reading frames (ORFs), gene boundaries were manually refined using MAFFT v7.481 (Katoh and Standley 2013; Katoh et al. 2019) by aligning sequences with homologous genes from related species. Introns and additional ORFs were predicted using MFannot (Lang et al. 2023).

### 2.3. Phylogenetic analyses

The amino acid sequences of 14 core mitochondrial PCGs were used for phylogenetic analysis. Each gene was aligned individually using MAFFT with the L-INS-i strategy and trimmed using TrimAl v1.4.rev15 (Capella-Gutiérrez et al. 2009) with the -gappyout option. Maximum likelihood (ML) phylogenetic inference was performed using IQ-TREE v2.4.0 (Minh et al. 2020) under a partition model (-spp option) with ModelFinder (Kalyaanamoorthy et al. 2017) selecting the best-fit models. Node support was assessed with 1,000 ultrafast bootstrap replicates (Hoang et al. 2018; Minh et al. 2013) and the SH-like approximate likelihood ratio test (Anisimova et al. 2011). Bayesian inference (BI) was also performed in MrBayes v3.2.7a (Ronquist et al. 2012) using a “mixed” amino acid model with partition-specific rates (ratepr = variable). Two Markov chain Monte Carlo (MCMC) runs were conducted for 1,000,000 generations with a 25% burn-in; convergence was confirmed by an average standard deviation of split frequencies < 0.01. The tree was visualized using iTOL v7 (Letunic and Bork 2024).

## 3. Results

**Figure 2.**
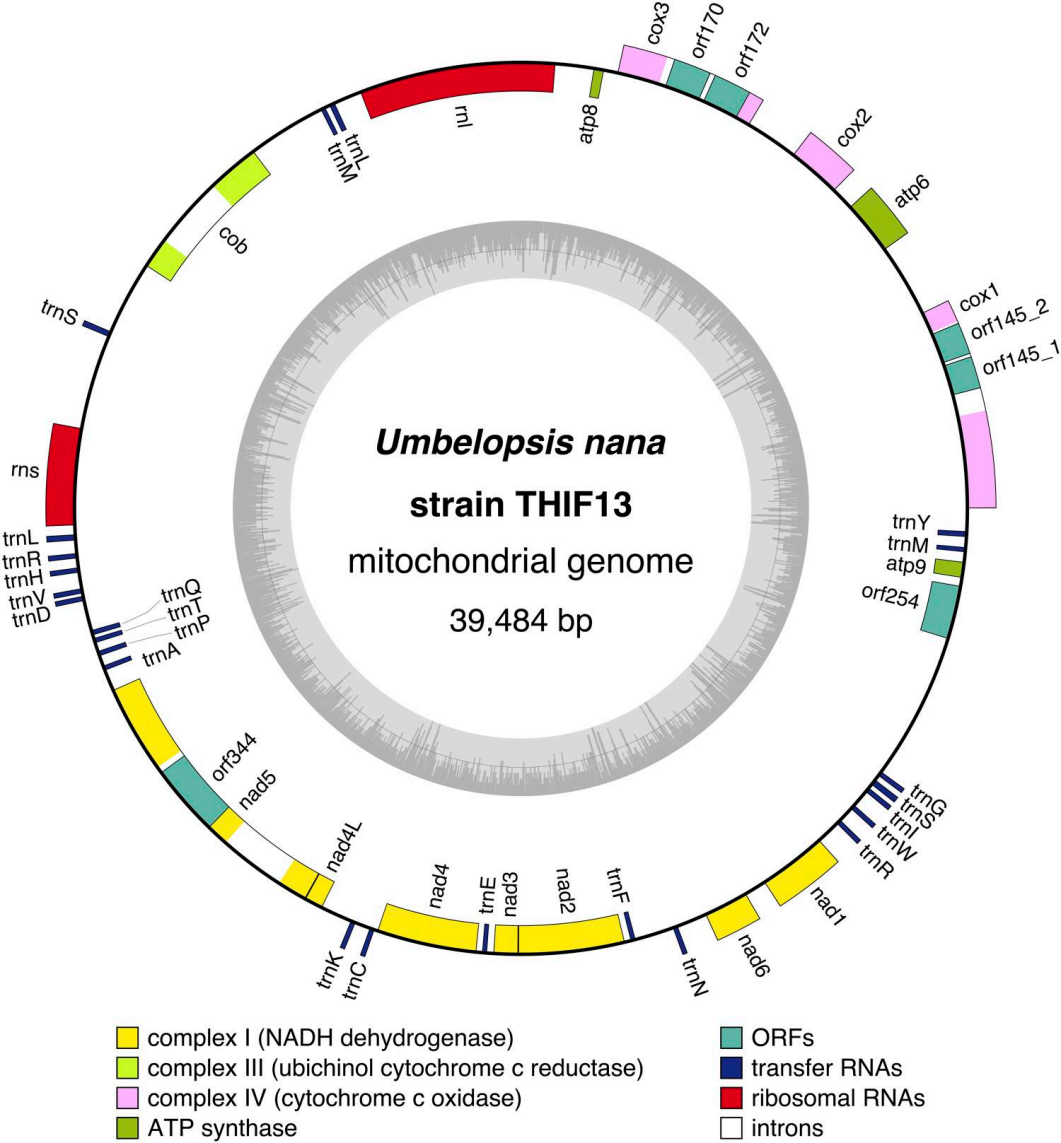
Circular mitochondrial genome map of *Umbelopsis nana* strain THIF13, generated with OrganellarGenomeDRAW (OGDRAW) v1.3.1 (Greiner et al. 2019). The inner circle shows the GC content, with the threshold line indicating the average GC content of the genome.

The complete mitochondrial genome of *U. nana* was assembled into a single circular molecule of 39,484 bp, with a GC content of 32.7% (Figure 2). The assembly was supported by a high mean sequencing depth of 1,139×, showing uniform coverage across the entire mitochondrial genome (Supplementary Figure S1). The genome contains 45 genes: 20 PCGs, two ribosomal RNA (rRNA) genes (*rnl* and *rns*), and 23 transfer RNA (tRNA) genes. Of the 20 PCGs, 14 were identified as core components of the oxidative phosphorylation system (*atp6*, *atp8*, *atp9*, *cob*, *cox1*, *cox2*, *cox3*, *nad1*, *nad2*, *nad3*, *nad4*, *nad4L*, *nad5*, and *nad6*). A comparison of the gene arrangement with other species in the Mucoromycotina showed that the gene order in *U. nana* is highly distinct, suggesting extensive genomic rearrangements. In addition, five Group I introns were identified within *cox1*, *cox3*, *cob* (one each), and *nad5* (two) (Supplementary Figure S2). These introns were named according to the standardized nomenclature for fungal mitochondrial introns (Zhang and Zhang 2019) (Supplementary Table S1). The remaining six PCGs were identified as homing endonuclease genes (HEGs), including five intronic HEGs belonging to the GIY-YIG or LAGLIDADG families and one intergenic HEG.

**Figure 3.**
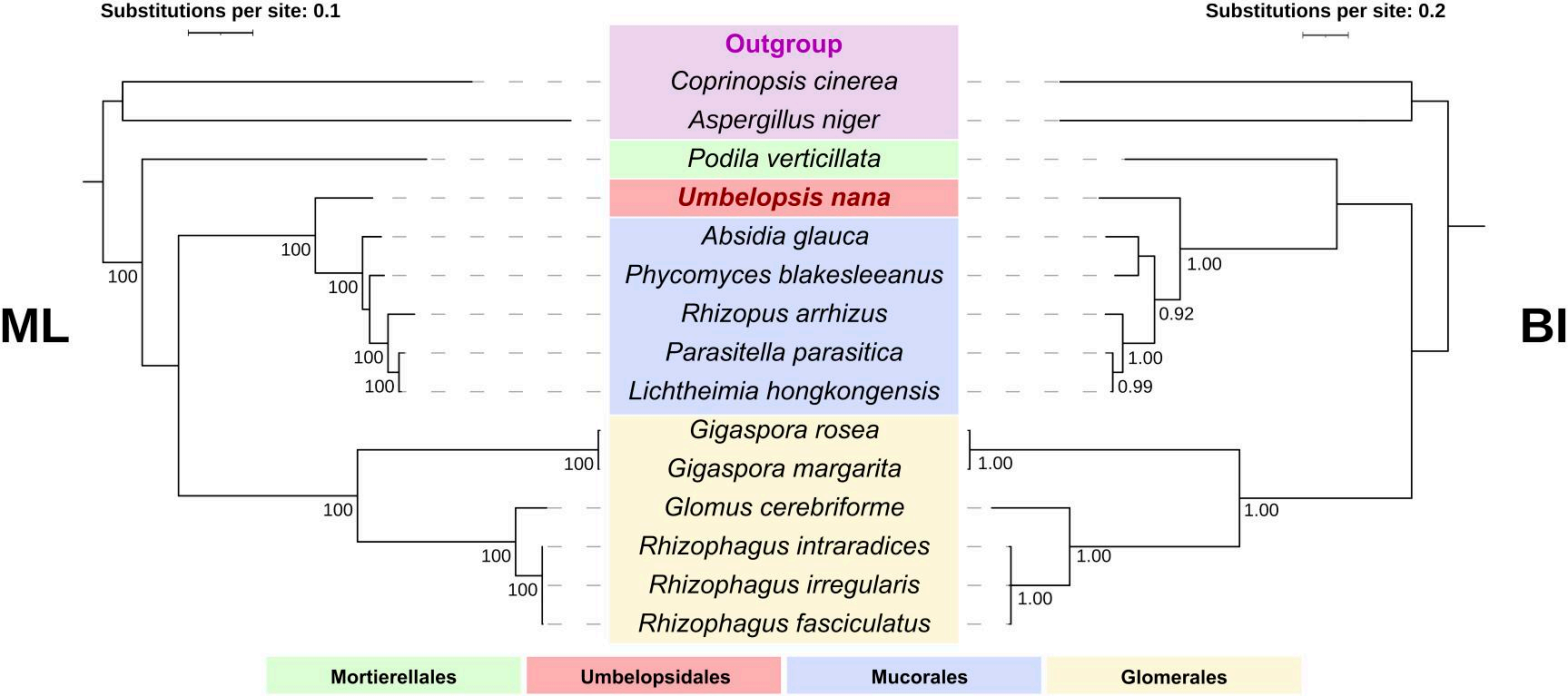
The phylogenetic positions of *U. nana* and related lineages. Trees were inferred from the concatenated amino acid sequences of 14 core mitochondrial PCGs (*atp6*, *atp8*, *atp9*, *cob*, *cox1*, *cox2*, *cox3*, *nad1*, *nad2*, *nad3*, *nad4*, *nad4L*, *nad5*, and *nad6*). (Left) ML tree calculated by IQ-TREE; nodal support was assessed using the ultrafast bootstrap (UFBoot) method with 1,000 replicates. The selected models included LG+F + G4 (for *atp6* and *nad4*), mtZOA + G4 (*atp8*, *atp9*, *cob*, *cox3*, *nad1*, *nad4L*, and *nad5*), mtZOA + I+R2 (*cox1*), LG+G4 (*cox2*), mtInv + F+G4 (*nad2*), and Q.yeast + G4 (*nad3* and *nad6*). (Right) BI tree calculated by MrBayes; the MCMC analysis was run for 1,000,000 generations. Numbers at the nodes indicate UFBoot values for the ML tree and Bayesian posterior probabilities (BPP) for the BI tree; only values with UFBoot > 70 and BPP > 0.90 are shown. *U. nana* is highlighted in red, and colored boxes indicate taxonomic groups as shown in the legend. The accession numbers of the sequences were as follows: *Absidia glauca* [NC_036158] (Ellenberger et al. 2016), *Lichtheimia hongkongensis* [NC_024200] (Leung et al. 2014), *Parasitella parasitica* [NC_024944] (Ellenberger et al. 2014), *Rhizopus arrhizus* [NC_006836] (Seif et al. 2005), *Gigaspora margarita* [NC_016684] (Pelin et al. 2012), *Gigaspora rosea* [NC_016985] (Nadimi et al. 2012), *Glomus cerebriforme* [NC_022144] (Beaudet et al. 2013), *Rhizophagus intraradices* [NC_012056] (Lee and Young 2009), *Rhizophagus fasciculatus* [NC_029185] (Nadimi et al. 2016), *Rhizophagus irregularis* [NC_014489] (Kokkoris et al. 2024), *Podila verticillata* [CM002878] (Seif et al. 2005), *Phycomyces blakesleeanus* [NC_027730] (Corrochano et al. 2016), *Coprinopsis cinerea* [NW_003307477] (Stajich et al. 2010), and *Aspergillus niger* [NC_007445] (Juhász et al. 2008).

Phylogenetic trees based on 14 core mitochondrial PCGs placed *U. nana* within the Mucoromycotina (Figure 3). Both ML and BI analyses resolved *U. nana* (Umbelopsidales) as a sister lineage to the Mucorales with maximal support (UFBoot = 100%, BPP = 1.00). In contrast to this stable placement, the deeper relationships among Mortierellomycotina, Glomeromycotina, and the Mucoromycotina clade were weakly supported and differed between the two methods. In the ML tree, Mortierellomycotina branched off first, though the subsequent branch uniting the remaining lineages was poorly supported (SH-aLRT = 6.1%, UFBoot = 52%). Conversely, the BI analysis placed Glomeromycotina as the basal-most lineage, with a posterior probability of 0.87 for this topology.

## 4. Discussion and conclusion

The *U. nana* mitochondrial genome (39,484 bp) is relatively compact within the Mucoromycotina. Its size is comparable to that of *L. hongkongensis* (31,830 bp; Leung et al. 2014) but substantially smaller than other Mucorales species, such as *P. parasitica* (83,361 bp), *A. glauca* (63,080 bp), *P. blakesleeanus* (62,082 bp), and *R. arrhizus* (54,178 bp). The number of introns corresponds closely to genome size, with *L. hongkongensis* and *U. nana* harboring only two and five introns, respectively. In contrast, *R. arrhizus*, *P. blakesleeanus*, *A. glauca*, and *P. parasitica* exhibit a stepwise increase in intron content, containing 9, 17, 20, and 24 introns, respectively. A similar trend is observed in their internal ORFs; while *L. hongkongensis* lacks intronic ORFs, *U. nana*, *R. arrhizus*, *P. blakesleeanus*, *A. glauca*, and *P. parasitica* harbor 5, 5, 12, 9, and 24 intronic ORFs, respectively. This suggests that mitochondrial genome evolution in this group is largely shaped by the proliferation of mobile genetic elements.

The phylogenetic analysis confirms the position of *U. nana* as a sister lineage to the Mucorales clade within the subphylum Mucoromycotina. The topological inconsistencies observed at deep nodes among Mortierellomycotina, Glomeromycotina, and Mucoromycotina likely reflect limited phylogenetic signals or rapid radiation among early-diverging fungi, as previously reported (James et al. 2006; Spatafora et al. 2016). This study provides a valuable genomic resource for the genus *Umbelopsis* and the order Umbelopsidales, contributing to a deeper understanding of the evolutionary patterns and diversity of mitochondrial genomes within the Mucoromycotina.

## Supplementary Material

Supplemental Material

## Data Availability

The genome sequence data that support the findings of this study are openly available in GenBank of NCBI at the Accession no. LC885050. The accession no. of BioProject, Bio-Sample, and DDBJ Sequence Read Archive (DRA) are PRJDB25744, SAMD00919913, and DRA021410, respectively.
