# Supplementary material for: The complete mitochondrial genome sequence of the saprotrophic filamentous fungus Umbelopsis nana
Source: Mitochondrial DNA B Resour. 2026 May 7;11(6):717–21. doi: 10.1080/23802359.2026.2668248 (PMC13159603; doi:10.1080/23802359.2026.2668248)
Supplement: Supplemental Material [file TMDN_A_2668248_SM0283.pdf]

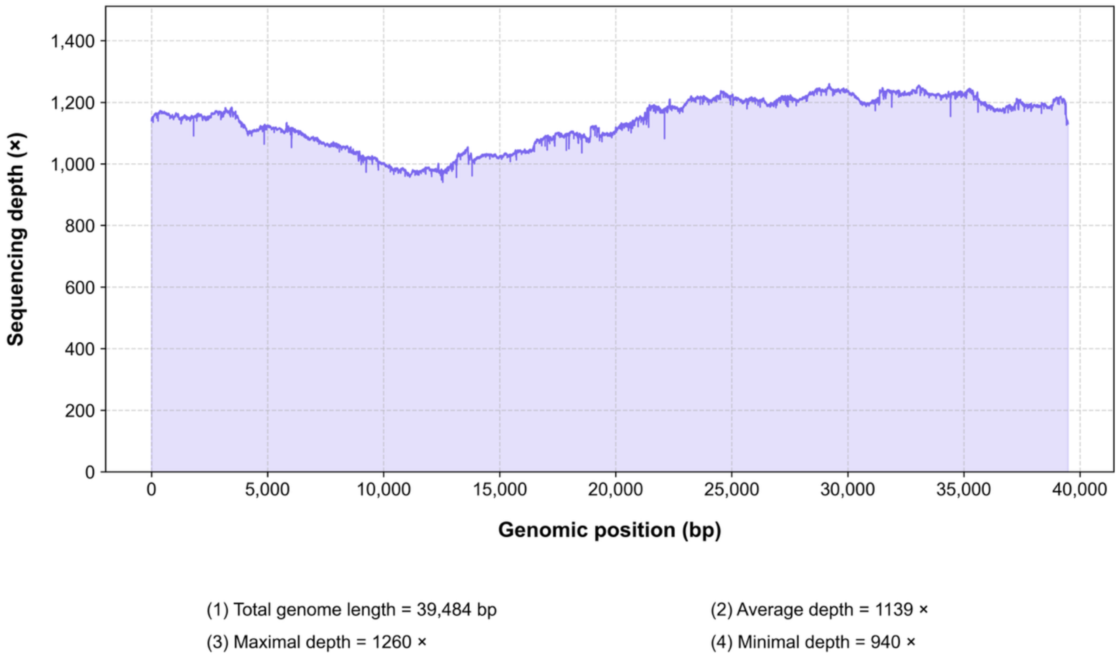

Figure S1 Sequencing depth and coverage map of *Umbelopsis nana* strain THIF13 mitochondrial genome. The plot was generated by mapping PacBio HiFi reads to the final genome assembly using minimap2 v2.30 (Li, 2018, 2021), followed by depth calculation at each base position with samtools v1.3.1 (Danecek et al., 2021).

For Review Only

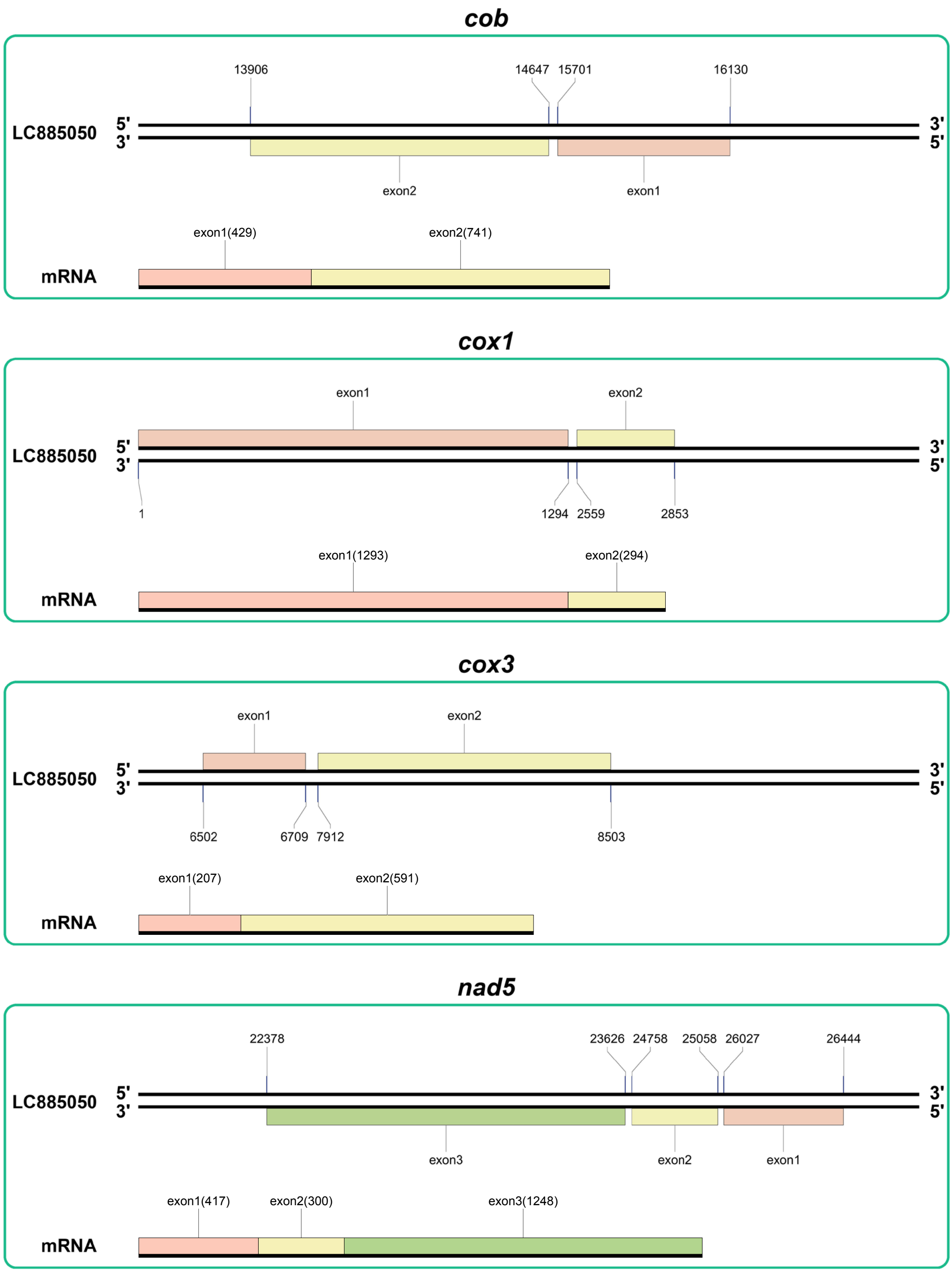

Figure S2 Cis-splicing genes of the *Umbelopsis nana* strain THIF13 mitochondrial genome, generated with Plant Mitochondrial Genomes Map (PMGmap) (Zhang et al., 2024). Numbers in brackets indicate the length of each exon in nucleotides (bp).

Table S1 Characteristics of introns and homing endonucleases in the *Umbelopsis nana* strain THIF13 mitochondrial genome.

Host Gene	Intron Name	Group	Intron Location (nt)	ORF Name	Domain / HEG Family	ORF Location (nt)
cox1	Una.cox1P1296	IB	1294..2558	*orf145_1*	GIY-YIG	1612..2049
				orf145_2	GIY-YIG	2086..2523
cox3	Una.cox3P219	IB	6709..7911	*orf172*	LAGLIDADG	6709..7227
				orf170	LAGLIDADG	7291..7803
cob	Una.cobP429	ID	14647..15700*	-	-	-
nad5	Una.nad5P417	IB	25058..26026*	-	-	-
	Una.nad5P717	IB	23626..24757*	orf344	LAGLIDADG	23723..24757*
Intergenic	-	-	-	*orf254*	GIY-YIG	37620..38384*

*Asterisks indicate features on the complementary strand.

For Peer Review Only

Supplemental material references

Danecek, P., Bonfield, J. K., Liddle, J., Marshall, J., Ohan, V., Pollard, M. O., Whitwham, A., Keane, T., McCarthy, S. A., Davies, R. M., & Li, H. (2021). Twelve years of SAMtools and BCFtools. *GigaScience*, *10*(2). https://doi.org/10.1093/gigascience/giab008

Li, H. (2018). Minimap2: pairwise alignment for nucleotide sequences. *Bioinformatics (Oxford, England)*, *34*(18), 3094–3100.

Li, H. (2021). New strategies to improve minimap2 alignment accuracy. *Bioinformatics (Oxford, England)*, *37*(23), 4572–4574.

Zhang, X., Chen, H., Ni, Y., Wu, B., Li, J., Burzyński, A., & Liu, C. (2024). Plant mitochondrial genome map (PMGmap): A software tool for the comprehensive visualization of coding, noncoding and genome features of plant mitochondrial genomes. *Molecular Ecology Resources*, *24*(5), e13952.

For Peer Review Only
